# CHA2DS2‐VASc score predicts atrial fibrillation recurrence after cardioversion: Systematic review and individual patient pooled meta‐analysis

**DOI:** 10.1002/clc.23147

**Published:** 2019-02-11

**Authors:** Francesco Vitali, Matteo Serenelli, Juhani Airaksinen, Rita Pavasini, Anna Tomaszuk‐Kazberuk, Elzbieta Mlodawska, Samuli Jaakkola, Cristina Balla, Lorenzo Falsetti, Nicola Tarquinio, Roberto Ferrari, Angelo Squeri, Gianluca Campo, Matteo Bertini

**Affiliations:** ^1^ Cardiovascular Center Azienda Ospedaliero‐Universitaria di Ferrara Ferrara Italy; ^2^ Heart Centre Turku University Hospital and University of Turku Turku Finland; ^3^ Department of Cardiology University Hospital in Bialystok Bialystok Poland; ^4^ Internal and Sub‐intensive Medicine Department A.O.U. “Ospedali Riuniti” Ancona Italy; ^5^ Department of Internal Medicine Ospedale 'S.S. Benvenuti e Rocco' Ancona Italy; ^6^ Maria Cecilia Hospital GVM Care and Research Cotignola Italy

**Keywords:** arrhythmia, atrial fibrillation, cardioversion, CHA2DS2‐VASc, recurrence

## Abstract

**Background:**

Despite progresses in the treatment of the thromboembolic risk related to atrial fibrillation (AF), the management of recurrences remains a challenge.

**Hypothesis:**

To assess if congestive heart failure or left ventricular systolic dysfunction (CHA_2_DS_2_‐VASc) score is predictive of early arrhythmia recurrence after AF cardioversion.

**Methods:**

Systematic review and individual patient pooled meta‐analysis following Preferred Reporting Items for Systematic reviews and Meta‐Analyses guidelines. Inclusion criteria: observational trials in patients with AF undergoing cardioversion, available data on recurrence of AF and available data on CHA_2_DS_2_‐VASc score. Clinical studies of interest were retrieved by PubMed, Cochrane Library, and Biomed Central. Seven authors were contacted for joining the patient level meta‐analysis, and three shared data regarding anthropometric measurements, risk factors, major comorbidities, and CHA_2_DS_2_‐VASc score. The primary outcome was the recurrence of AF after cardioversion in patients free from antiarrhythmic prophylaxis. Univariate and multivariate logistic regression was performed.

**Results:**

Overall, we collect data of 2889 patients: 61% were male, 50% with hypertension, 12% with diabetes, and 23% with history of ischemic heart disease. The median CHA2DS2‐VASc score was 2.. At the multivariate analysis, chronic kidney disease (odds ratio [OR] 1.94; 95% confidence interval [CI] 1.12‐3.27; *P* = 0.01), peripheral artery disease (OR 1.65; 95% CI 1.23‐2.19; *P* < 0,0001), previous use of beta blockers (OR 1.5; 95% CI 1.19‐1.88; *P* < 0.0001), and CHA2DS2‐VASc score > 2 (OR 1.37; 95% CI 1.1‐1.68; *P* = 0.002) were independent predictors of early recurrence of AF.

**Conclusions:**

CHA2DS2‐VASc score predicts early recurrence of AF in the first 30 days after electrical or pharmacological cardioversion.

**Protocol registration**

PROSPERO (CRD42017075107).

## 1 INTRODUCTION

Atrial fibrillation (AF) is the most frequent arrhythmia causing a considerable burden of mortality and morbidity in developed country.^1^ Despite progresses aimed to reduce the risk of stroke by new oral anticoagulants,^2^, ^3^, ^4^ AF management remains somehow problematic because of the difficulty to predict recurrences that reduce quality of life, and increase hospital admissions.^1^

Although trials comparing rhythm to rate control (with appropriate anticoagulation) resulted in neutral clinical outcomes,^5^, ^6^, ^7^, ^8^, ^9^, ^10^, ^11^, ^12^, ^13^ a rhythm control strategy is currently considered the first option in patients with symptomatic AF, and in young patients with a first episode of the arrhythmia.^1^

In paroxysmal AF, recurrent episodes are mainly due to factors triggering arrhythmias (triggers), whereas perpetuating factors (perpetrators) are the key elements in persistent and permanent AF.^14^, ^15^, ^16^ Among perpetrators, different factors are considered as independent predictors for the reoccurrence of AF, such as advanced age, heart failure (HF), previous myocardial infarction (MI), hypertension, 17, diabetes, obesity,^18^ presence of valvular heart disease,^19^ chronic obstructive pulmonary disease (COPD),^19^, ^20^ and cigarette smoking.^21^ Those stressors induce a time‐dependent maladaptive cascade of events with a progressive atrial structural and electrical remodeling leading to the development and maintenance of AF.

The CHA_2_DS_2_‐VASc (congestive heart failure or left ventricular systolic dysfunction, hypertension; age ≥ 75 years; diabetes mellitus; prior stroke or transient ischemic attack (TIA) or thromboembolism; vascular disease; age 65‐74 years; female sex), a score which predicts the risk of ischemic event, in patients with AF,^22^ is currently considered the cornerstone for the management of anticoagulation therapy. Because of the high consistency in the quantification of the thromboembolic risk, this score has been studied in different settings and not only in AF patients. Indeed, variables included in the CHA_2_DS_2_‐VASc score are associated with the risk of stroke in patients without AF but affected by acute coronary syndrome.^23^ Furthermore, some authors observed that an increasing CHA_2_DS_2_‐VASc was associated to an increased rate of high atrial rate responses in a population without previous diagnosis of AF, and thus with an increased probability of developing atrial arrhythmias.^24^ Saliba et al underlined that higher CHA_2_DS_2_‐VASc scores were directly associated with new‐onset of AF.^25^ All these studies suggest a potential role of CHA_2_DS_2_‐VASc as a marker of atrial electrical or mechanical remodeling, which could be responsible of AF recurrences after cardioversion. However, its predictive value for recurrences of AF is controversial.^26^, ^27^, ^28^, ^29^, ^30^, ^31^, ^32^

Thus, we performed a systematic review and individual patient pooled meta‐analysis to assess the value of the CHA_2_DS_2_‐VASc score as a predictor of early AF recurrence after successful cardioversion.

## 2 METHODS

### 2.1 Search strategy

We performed a systematic review and meta‐analysis following Preferred Reporting Items for Systematic reviews and Meta‐Analyses (PRISMA) amendment to the Quality of Reporting of Meta‐analyses (QUOROM) statement.^33^, ^34^, ^35^, ^36^ Details of the protocol for this meta‐analysis are registered on PROSPERO with identifier CRD42017075107. The search strategy was elaborated by FV in July 2017. The terms searched were ([CHA2*] AND [cardioversion]) AND ([recurrence] OR [reoccurrenc]) OR [AF] OR [atrial fibrillatio]) OR [atrial flutte]) OR [flutte]) OR [strok]) OR [ictus]). The databases analyzed were Google Scholar, PubMed, and Biomed Central and Cochrane library. Only papers published in English and in peer‐reviewed journal were selected. Two independent reviewers (MS, FV) analyzed the records and decided those deserving a full‐text analysis. The same reviewers (MS, FV) independently analyzed references of all the evaluated articles to include papers not found with the database search strategy. Disagreement was solved with consensus.

### 2.2 Selection criteria

The inclusion criteria for the studies were: (a) observational or interventional trials in patients with AF or atrial flutter undergoing cardioversion, (b) available data about recurrence of AF or atrial flutter, (c) available data on CHA_2_DS_2_‐VASc score.

Exclusion criteria were: (a) duplicate reports, (b) duplicate of the sample size, (c) case reports/series, (d) review papers, and (e) lack of outcome of interest.

### 2.3 Data abstraction, endpoints, contact with authors

After the selection of the papers, the reviewers (FV, MS) completed a database with data regarding: the journal, year of publication, the hospital center, population characteristics, CHA_2_DS_2_‐VASc, and outcome of interest. The primary outcome of the study was the recurrence of AF after electrical or pharmacological cardioversion. Next, each corresponding author of the papers selected were asked to fill a patient‐level database with the following data: age, sex, height (cm), weight (kg), body mass index , major cardiac disease, cardiovascular risk factors, comorbidities (COPD, previous stroke or TIA, chronic kidney disease (glomerular filtration rate < 60); peripheral artery disease (PAD)); previous myocardial infarction, previous percutaneous coronary intervention, previous coronary artery bypass grafting; use of novel oral anticoagulants; use of oral anticoagulants; cardiovascular therapy (antiplatelets; beta‐blockers; angiotensin‐converting enzymes inhibitors /angiotensin II receptor blockers ; statins); starting electrocardiography (ECG) rhythm; type of cardioversion (electrical or pharmacological); drug used for cardioversion (if pharmacological); ECG rhythm after cardioversion attempt (sinus rhythm or AF); antiarrhythmic drugs; recurrence of AF or flutter in first 30 days; early recurrence of AF (first 24 hours after cardioversion); recurrence of AF or flutter (days from cardioversion). Data were assessed in a consistent manner across all studies with standard definitions and parameters. In patients with more AF recurrences only the first episode was considered in the analysis.

### 2.4 Quality assessment

Two unblinded reviewers (RP, MS) independently evaluated the quality of the included studies using a modified version of the Newcastle‐Ottawa Scale (NOS) for cohort studies^37^ (Table S1, Supporting Information), excluding the analysis of the section “Comparability” and question 2 of the section “Selection” (“selection of the non‐exposed cohort”). Discrepancies between reviewers have been solved by consensus. No study was excluded on the basis of this analysis. The maximum score obtained was 6 Table S1.

### 2.5 Data analysis and synthesis

Demographics and other baseline characteristics were summarized in terms of mean ± SD if with normal distribution, otherwise as median and interquartile range. Continuous variables were evaluated for normal distribution with Kolmogorov‐Smirnov test. Categorical variables were expressed as number and percentage (%). Variables were compared between patients with and without recurrence of AF, using the *t* test for independent group, the *χ* ^2^ test and the Mann‐Whitney *U* test as appropriate, and a *P*‐value of 0.05 was considered to be statistically significant.

Univariate logistic regression was performed to evaluate the relationship between the baseline population characteristics and the primary outcome (Table S3), those statistically significant (*P* < 0.05) were entered into a multivariate model for assessing the relation with primary outcome, excluding variables already included in the CHA_2_DS_2_‐VASc score. The multivariate analysis was performed in two models, in the first one considering CHA_2_DS_2_‐VASc score as continuous variable and in the second one considering it as a dichotomized variable (<2 vs ≥2, as per usual interpretation of CHA_2_DS_2_‐VASc score). Odds ratios (OR) and 95% confidence intervals (CI) were calculated. To establish the predictive value of CHA_2_DS_2_‐VASc score, receiver operating characteristics (ROC) curve with area under the curve was also calculated for the primary outcome. Using Youden index (J), the best cut‐off (c) point for grip strength was obtained, by the formula *J* = max_*c*_ {sensibility (*c*) + specificity (*c*) − 1}. The OR for the relation between AF recurrence and the primary outcome were calculated for each single study and then pooled using the Mantel Haenszel method, using a random‐effects method, and the generic inverse variance approach.^38^ The weight of the individual studies was measured as the inverse of the estimated variance of the log OR,^38^, ^39^ and heterogeneity across the trials has been assessed using the *I* ^2^ statistics, with a value of 0% to 24.9% considered insignificant, 25% to 49.9% mild, 50% to 74.9% moderate and ≥75% considered severe.^44^ Publication bias was appraised by Begg and Mazumdar rank correlation.^38^ Prometa software 3 (Internovi, Cesena, Italy), RevMan 5 (The Cochrane Collaboration, The Nordic Cochrane Centre, Copenhagen, Denmark) and SPSS version 24 (IBM, Italy) were the software used for statistical analyses.

## 3 RESULTS

### 3.1 Search strategy

A total of 2408 records were analyzed. After the first evaluation of titles and abstracts, 22 studies were screened and of these 15 were excluded with reasons as reported in Figure S1, while the remaining seven were analyzed as full‐paper.^26^, ^27^, ^28^, ^29^, ^30^, ^31^, ^32^ Thereafter the seven corresponding authors were contacted for joining the patient‐level meta‐analysis. Three authors agreed to share data and were included in qualitative and quantitative analysis^26^, ^27^, ^28^ (Figure S1 and Table S1).

### 3.2 Patient level meta‐analysis

#### 3.2.1 Population characteristics

**Figure 1:**
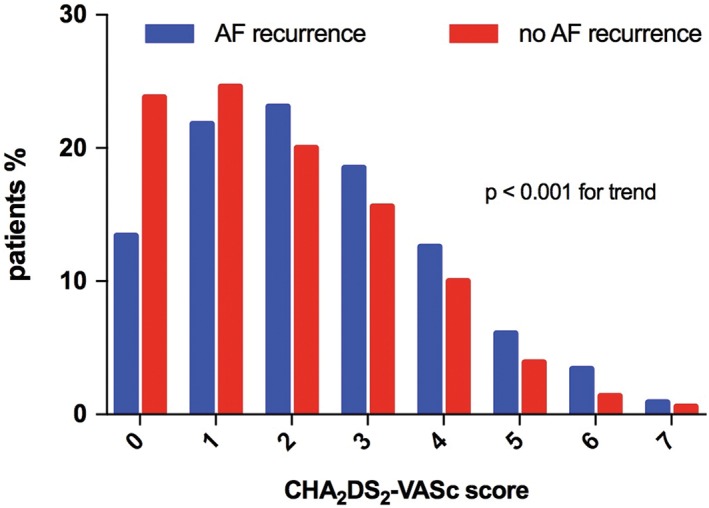
CHA2DS2‐VASc distribution between groups. Vertical line: % CHA2DS2‐VASc distribution; horizontal line: CHA2DS2‐VASc scores. AF, atrial fibrillation

The initial study population involved 5861 patients. After exclusion of patients (a) without AF recurrence data (n = 501), (b) with ineffective cardioversion in restoring sinus rhythm (n = 422), (c) taking antiarrhythmic drugs after cardioversion for rhythm control strategy (n = 1748), and (d), those without data on antiarrhythmic drug therapy (n = 301), 2889 patients were included in the final analysis. The follow up ranged between 7 and 30 days after the cardioversion. The mean age was 62.86 ± 13.17 years, 39.3% were female; 49.7% of the patients had hypertension, 11.6% diabetes, 6.7% history of previous stroke/TIA (Table 1). The median of CHA_2_DS_2_‐VASc score was 2.^1^, ^2^, ^3^ Overall 21,7% of the patients had a CHA_2_DS_2_‐VASc score of 0; 24.1% of the patients had a CHA_2_DS_2_‐VASc score of 1 and 20.7% of 2 and 33.5% had a CHA_2_DS_2_‐VASc score greater than or equal to 3. The distribution of CHA_2_DS_2_‐VASc score according to the presence of recurrence of arrhythmia is shown in Figure 1.

**Table 1:** Baseline population characteristics

	Overall population (n = 2889)	No AF recurrence (n = 2301)	AF recurrence (n = 588)	*P‐*value
Age mean ± SD	62.86 ± 13.17	62.14 ± 13.39	65.66 ± 11.88	**<0.0001**
Age ≥65 and <75, n (%)	847 (29.3)	657 (28.6)	190 (32.3)	0.07
Age ≥75, n (%)	568 (19.7)	426 (18.5)	142 (24.1)	**0.002**
Female, n (%)	1135 (39.3)	881 (38.3)	254 (43.2)	**0.03**
Ischemic heart disease, n (%)	661 (22.9)	488 (21.2)	173 (29.4)	**<0.0001**
Hypertension, n (%)	1435 (49.7)	1105 (48)	330 (56.1)	**<0.0001**
Diabetes, n (%)	336 (11.6)	255 (11.1)	81 (13.8)	0.069
Previous stroke/TIA, n (%)	194 (6.7)	138 (6)	56 (9.5)	**0.002**
Chronic kidney disease, (GFR < 60 mL/min) n (%)	58 (2%)	35 (1.5)	23 (3.9)	**<0.0001**
PAD, n (%)	278 (9.6)	193 (8.4)	85 (14.5)	**<0.0001**
Previous, MI n (%)	347 (12)	253 (11)	94 (16)	**0.001**
Vascular disease, n (%)	574 (19.9)	412 (17.9)	162 (27.6)	**<0.0001**
Congestive heart failure, n (%)	189 (6.5)	110 (4.8)	79 (13.4)	**<0.0001**
OACs, n (%)	734 (25.4)	472 (20.5)	262 (44.6)	**<0.0001**
Antiplatelets, n (%)	955 (33.1)	746 (32.4)	209 (35.5)	0.151
Beta‐blockers, n (%)	1867 (64.6)	1450 (63)	417 (70.9)	**<0.0001**
CHA2DS2‐VASc score, SCU	1.94 ± 1.59	1.84 ± 1.56	2.32 ± 1.64	**<0.0001**
CHADS2 score, SCU	1.01 ± 1.06	0.94 ± 1.03	1.27 ± 1.13	**<0.0001**
CHA2DS2‐VASc score ≥2 n (%)	1568 (54.3)	1187 (51.6)	381 (64.8)	**<0.0001**

Abbreviations: AF, atrial fibrillation; CHA2DS2‐VASc, Congestive heart failure or Left ventricular systolic dysfunction; GFR, glomerular filtration rate; MI, myocardial infarction; OAC, oral anticoagulant; PAD, peripheral artery disease; SCU, single change unit; TIA, transient ischemic attack.

Hypertension; Age ≥ 75 years; Diabetes Mellitus; Prior Stroke or TIA or thromboembolism; Vascular disease; Age 65‐74 years; female sex. CHADS2: Congestive heart failure or Left ventricular systolic dysfunction, Hypertension; Age ≥ 75 years; Diabetes Mellitus; Prior Stroke or TIA or thromboembolism.

#### 3.2.2 Primary outcome

Results of the univariate analysis are summarized in Table S3. Besides having higher incidences of the single variables already included in CHA_2_DS_2_‐VASc score (Table S3), patient with recurrence of AF were older (65.66 ± 11.88 vs 62.14 ± 13.39; *P* < 0.0001**;** OR 1.02; 95% CI 1.01‐1.03), higher use of oral anticoagulants (OACs) (44.6% vs 20.5%; *P* < 0,0001; OR 2.95; 95% CI 2.43‐3.55) and beta‐blockers (70.9% vs 63%; *P* < 0.0001; OR 1.75; 95% CI 1.38‐2.19). Moreover, the prevalence of chronic kidney disease (CKD), defined as GRF < 60 mL/min, was also higher in patients with AF recurrence (1.5% vs 3.9%; *P* **<** 0.0001**;** OR 2.58; 95% CI 1.52‐4.27). CHA_2_DS_2_‐VASc score considered as a continuous variable was related to an increased risk of recurrence of AF (*P* < 0.0001; OR 1.19; 95% CI 1.12‐1.25). After multivariate analysis, only PAD (*P* < 0.0001; OR 1.6; 95% CI 1.22‐2.17), previous use of beta‐blockers (*P* < 0.0001; OR 1.5; 95% CI 1.18‐1.88) chronic kidney disease (p 0.015; OR 1.9; 95% CI 1.11‐3.24) and CHA_2_DS_2_‐VASc score (*P* < 0.0001; OR 1.13; 95% CI 1.06‐1.2) were independent predictive variables of the recurrence of AF (Table 2). These findings were confirmed also in the multivariate model considering CHA_2_DS_2_‐VASc score as a dichotomous variable with cutoff at ≥2 (*P* < 0.002; OR 1.37; 95% CI 1.1‐1.68) (Table 2).

**Table 2:** Multivariate logistic regression for AF recurrence

	CHA2DS2‐VASc score SCU			CHA2DS2‐VASc ≥ 2		
	OR	95% CI	*P*	OR	95% CI	*P*
Ischemic disease	1.12	0.88‐1.4	0.345	1.16	0.92‐1.45	0.19
Chronic kidney disease (GRF < 60 mL/min)	1.9	1.11‐3.24	**0.015**	1.94	1.12‐3.27	**0.01**
PAD	1.6	1.22‐2.17	**<0.0001**	1.65	1.23‐2.19	**<0.0001**
Beta‐blockers	1.5	1.18‐1.88	**<0.0001**	1.5	1.19‐1.88	**<0.0001**
CHA2DS2‐VASc	1.13	1.06‐1.2	**<0.0001**	1.37	1.1‐1.68	**0.002**

Abbreviations: CHA2DS2‐VASc: Congestive heart failure or Left ventricular systolic dysfunction; GFR: glomerular filtration rate; PAD: peripheral artery disease**.**

Hypertension; Age ≥ 75 years; Diabetes Mellitus; Prior Stroke or TIA or thromboembolism; Vascular disease; Age 65‐74 years; female sex.

The area under the ROC curve for CHA_2_DS_2_‐VASc and AF recurrence was 0.6 (95%CI 0.56‐0.60, *P* < 0.0001) (Figure S2). After the application of Youden index, the best cut‐off point for CHA_2_DS_2_‐VASc score was confirmed to be 2 (64.8% sensitivity, 48.4% specificity).

### 3.3 Study level meta‐analysis

**Figure 2:**
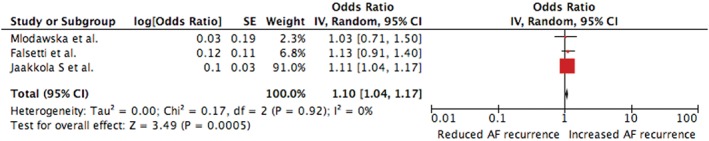
Forrest plot of the relation between CHA2DS2‐VASc (as ordinal variable) and atrial fibrillation recurrences. Data are displayed as odds ratio (95% CI). CI, confidence interval

**Figure 3:**
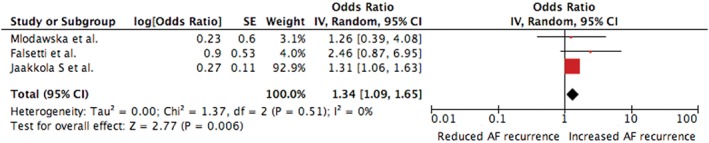
Forrest plot of the relation between CHA2DS2‐VASc ≥2 (as nominal variable) and atrial fibrillation recurrences. Data are displayed as odds ratio (95% CI). CI, confidence interval

The pooled OR of the predictive value of AF recurrences of CHA_2_DS_2_‐VASc score was 1.10 (95% CI 1.04‐1.17, *I* ^*2*^ 0%) and 1.34 (95% CI 1.09‐1.65, *I* ^*2*^ 0%) for CHA_2_DS_2_‐VASc score ≥2 (Figures 2 and 3). The analyses for publication bias were negative (*Z value for Kendall's tau* 0.52 and with *P* 0.602 for CHA_2_DS_2_‐VASc score as continuous variable and ≥2).

## 4 DISCUSSION

In addition to the well‐established ability to predict thromboembolic risk, our individual patient pooled meta‐analysis shows that CHA_2_DS_2_‐VASc score predicts also the risk of AF recurrence after electrical or pharmacological cardioversion in a common clinical low thromboembolic risk population (median of CHA_2_DS_2_‐VASc score = 2^1^, ^2^, ^3^). Furthermore, CHA_2_DS_2_‐VASc score, considered both as continuous and dichotomous variable (with a cut‐off value of ≥2), has proved to be an independent predictor of early recurrence of AF/atrial flutter after electrical or pharmacological cardioversion. The ROC curve analysis showed that a CHA_2_DS_2_‐VASc score ≥2 was linked to a 37% increase in risk of recurrence of arrhythmia. This finding could be related to the ability of the score to indirectly quantify complex pathophysiological substrates and modifiers responsible for AF. Patients with high CHA_2_DS_2_‐VASc score are exposed to several factors recognized as perpetrators of the arrhythmia, which induce maladaptive changes at a cellular and extracellular level leading to a more favorable substrate for the permanence and the recurrence of AF despite cardioversions.

We also identified chronic kidney disease (GRF < 60 mL/min) as an important predictor of early recurrence of AF in our population. Prevalence of AF is usually higher in patients with chronic renal impairment probably because of the increased sympathetic tone and renin‐angiotensin‐aldosterone system activation which, in turn, cause atrial electrical and structural remodeling.^40^ The predictive value of the score for risk of AF recurrence after cardioversion in patients with chronic kidney disease, however, has never been reported before.

PAD also was found to be a predictor of early recurrence of AF.^45^ PAD is known to be linked with an increased risk of AF but the mechanisms underlining are not fully understood.^41^ AF and PAD share risk factors and common pathophysiological pathways like increased inflammation levels, endothelial dysfunction, and a prothrombotic state.^41^

Finally, also the previous use of beta‐blockers resulted to be a predictor of early recurrence of AF. This was unexpected even if it may be explained by the tendency of clinicians to prescribe beta‐blockers as part of the treatment for heart failure.

The strength of our results came from the analysis of a large courts of patients, the high quality of data obtained, confirmed by the relation between CHA_2_DS_2_‐VASc and AF recurrence present both at a study level and at a patient level meta‐analysis. Thus, cut‐off value of CHA_2_DS_2_‐VASc score ≥2 could be used to estimate the risk of AF recurrences after electrical or pharmacological cardioversion in patients, with PAD and CKD.

In everyday clinical practice, as suggested by international guidelines^1^, ^42^ CHA_2_DS_2_‐VASc score should be calculating in every single patient with AF to predict the thromboembolic risk. Our findings suggest an additional value of the score: it should be considered in the decision‐making process for cardioversion as CHA_2_DS_2_‐VASc score will provide estimation of the likelihood of recurrences without the need of further exams and analysis.

The cut‐off value of CHA_2_DS_2_‐VASc score ≥2 may identify patients at higher risk of AF recurrences after cardioversion, in particular with comorbidities like PAD or CKD. New studies might be necessary to test if the addition of these two variables to the CHADsVASC could increase the sensitivity and specificity of the score on AF recurrences. Therefore, in patients with a CHA_2_DS_2_‐VASc score ≥2, if a rhythm control strategy is opted, it is reasonable to initiate antiarrhythmic prophylaxis after cardioversion.^8^, ^43^ Alternatively, a catheter ablation of the arrhythmic substrate could be considered.^43^ Finally, also, the setting of the cardioversion has to be analyzed; as a matter of fact our findings are useful in hemodynamically stable patients with AF. In an acute setting with acute instable patients cardioversion cannot be postponed and the trigger of the arrhythmia must be identified and treated.

### 4.1 Study limitations

This is a meta‐analysis and data are obtained retrospectively by each corresponding author; thus, bias related to incomplete data reporting cannot be excluded. Complete information regarding some variables, such as smoking habit, COPD, dyslipidemia, and ECG (eg, signs of atrial enlargement as atrial diameter or area) are lacking. We also had incomplete data on the use of renin‐angiotensin inhibitors and statins, which could have lowered the chance of recurrence of the arrhythmias.

The vast majority of the patients included in the meta‐analysis were enrolled in the study of Jaakkola et al^28^ Nevertheless, the analysis of *I* ^*2*^ disclosed the absence of heterogeneity (*I* ^*2*^ = 0). The multivariate analysis in each single study was lightly modified, based on the clinical variables available for each single study.

## 5 CONCLUSIONS

The CHA_2_DS_2_‐VASc score could be useful to predict early recurrence of AF/atrial flutter in the first 30 days after cardioversion.

## CONFLICTS OF INTEREST

Francesco Vitali and Matteo Serenelli are the guarantors of the content of the manuscript, including the data and analysis. Rita Pavasini, Matteo Bertini, Gianluca Campo, Cristina Balla: conception, design, analysis and interpretation of data. Juhani Airaksinen, Anna Tomaszuk‐Kazberuk, Elzbieta Mlodawska, Samuli Jaakkola, Lorenzo Falsetti, Nicola Tarquinio, Francesco Vitali, Matteo Serenelli: collection of data. Francesco Vitali, Matteo Serenelli, Rita Pavasini, Angelo Squeri: data analysis and interpretation. All authors: drafting of the manuscript and revising it critically for important intellectual content. All authors: final approval of the manuscript submitted.

## Supporting information


**Table S1**. New Castle Ottawa scale for quality assessment of paper included in the meta‐analysis
**Table S2**. Studies included in the meta‐analysis
**Table S3**. Univariate logistic regression
**Figure S1**. PRISMA flow‐chart.
**Figure S2**. CHA2DS2‐VASc score as predictor of atrial fibrillation recurrence—receiver operating characteristic curve
