# Supplementary material for: CHA2DS2‐VASc score predicts atrial fibrillation recurrence after cardioversion: Systematic review and individual patient pooled meta‐analysis
Source: Clin Cardiol. 2019 Feb 11;42(3):358–64. doi: 10.1002/clc.23147 (PMC6712331; doi:10.1002/clc.23147)

**Supplemental online material**

**Table 1s page 2**

**Table 2s page 3**

**Table 3s page 4**

**Figure 1s page 5**

**Figure 2s page 6**

**Table 1s : New Castle Ottawa scale for quality assessment of paper included in the meta-analysis**

| **References** | **NOS score (maximum 6)** | **Selection 1** | **Selection 3** | **Selection 4** | **Outcome 1** | **Outcome 2** | **Outcome 3** |
| --- | --- | --- | --- | --- | --- | --- | --- |
| *Jaakkola et al.* | 6 | a* | a* | a* | b* | a* | a* |
| *Falsetti et al.* | 6 | a* | a* | a* | b* | a* | a* |
| *Mlodawka et al.* | 6 | a* | a* | a* | b* | a* | a* |

*: value corresponding to 1 point for score

Because of the design of the studies, we decided to use the NOS scale for cohort studies, but not considering the section for “Comparability” and question 2 in the section “Selection” (“selection of the non exposed cohort”). Letters indicate the answer to questions as indicated in the original form of the NOS scale for cohort studies. For the full version of the NOS scale : <http://www.ohri.ca/programs/clinical_epidemiology/oxford.asp>.

**Table 2s : Studies included in the meta-analysis**

| **References** | **Year of publication** | **Number of patients** | **Geographic area** | **Time of the enrollement** | **Source for documentation of events** | **Type of cardioversion** | **Follow-up** |
| --- | --- | --- | --- | --- | --- | --- | --- |
| *Jaakkola et al.* | 2016 | 2602 | Finland | NA | - Medical records | - Electrical | 30 days |
| *Falsetti et al.* | 2014 | 219 | Italy | NA | - Medical records | -Pharmacological and electrical | 5 days after discharge |
| *Mlodawska et al.* | 2016 | 68 | Poland | January 2012 -April 2016 | - Medical records | - Electrical | 30 days |

**Table 3s: Univariate logistic regression**

| **AF recurrence** | | | |
| --- | --- | --- | --- |
|  | *OR* | *95% CI* | *p* |
| **Age mean** | **1,02** | **1,01-1,03** | **< 0,0001** |
| Age ≥65 & <75 | 1,19 | 0,97-1,43 | 0,07 |
| **Age ≥75** | **1,38** | **1,11-1,69** | **0,002** |
| **Female** | **1,21** | **1,01-1,43** | **0,03** |
| **Ischemic heart disease** | **1,52** | **1,25-1,83** | **< 0,0001** |
| **Hypertension** | **1,37** | **1,15-1,62** | **< 0,0001** |
| Diabetes | 1,27 | 0,96-1,64 | 0,069 |
| **Previous Stroke/TIA** | **1,61** | **1,15-2,21** | **0,002** |
| **Chronic kidney disease (GRF < 60 ml/min)** | **2,58** | **1,52-4,27** | **< 0,0001** |
| **PAD** | **1,78** | **1,36-2,32** | **< 0,0001** |
| **Previous MI** | **1,5** | **1,17-1,91** | **0,001** |
| **Vascular disease** | **1,7** | **1,37-2,09** | **< 0,0001** |
| **Congestive heart failure** | **2,92** | **2,18-3,87** | **< 0,0001** |
| **OACs** | **2,95** | **2,43-3,55** | **< 0,0001** |
| Antiplatelets | 1,14 | 0,94-1,38 | 0,151 |
| **Beta-blockers** | **1,75** | **1,38-2,19** | **< 0,0001** |
| **CHA2DS2-VASc score SCU** | **1,19** | **1,12-1,25** | **< 0,0001** |
| **CHADS2 score SCU** | **1,29** | **1,19-1,39** | **< 0,0001** |
| **CHA2DS2-VASc score ≥ 2 n°(%)** | **1,69** | **1,39-2,03** | **< 0,0001** |

**AF: atrial fibrillation; SD: standard deviation; MI: myocardial infarction; PAD: peripheral artery disease; OAC: oral anticoagulant; TIA: transient ischemic attack; GFR: glomerular filtration rate; SCU: single change unit; CHA2DS2-VASc: Congestive heart failure or Left ventricular systolic dysfunction,**[**Hypertension**](https://en.wikipedia.org/wiki/Hypertension)**;  Age ≥75 years; Diabetes Mellitus; Prior** [**Stroke**](https://en.wikipedia.org/wiki/Stroke) **or** [**TIA**](https://en.wikipedia.org/wiki/Transient_ischemic_attack) **or** [**thromboembolism**](https://en.wikipedia.org/wiki/Thromboembolism)**; Vascular disease; Age 65–74 years; female sex. CHADS2: Congestive heart failure or Left ventricular systolic dysfunction,**[**Hypertension**](https://en.wikipedia.org/wiki/Hypertension)**;  Age ≥75 years; Diabetes Mellitus; Prior** [**Stroke**](https://en.wikipedia.org/wiki/Stroke) **or** [**TIA**](https://en.wikipedia.org/wiki/Transient_ischemic_attack) **or** [**thromboembolism**](https://en.wikipedia.org/wiki/Thromboembolism)**.**

**Figure 1s: PRISMA flow-chart.**

**
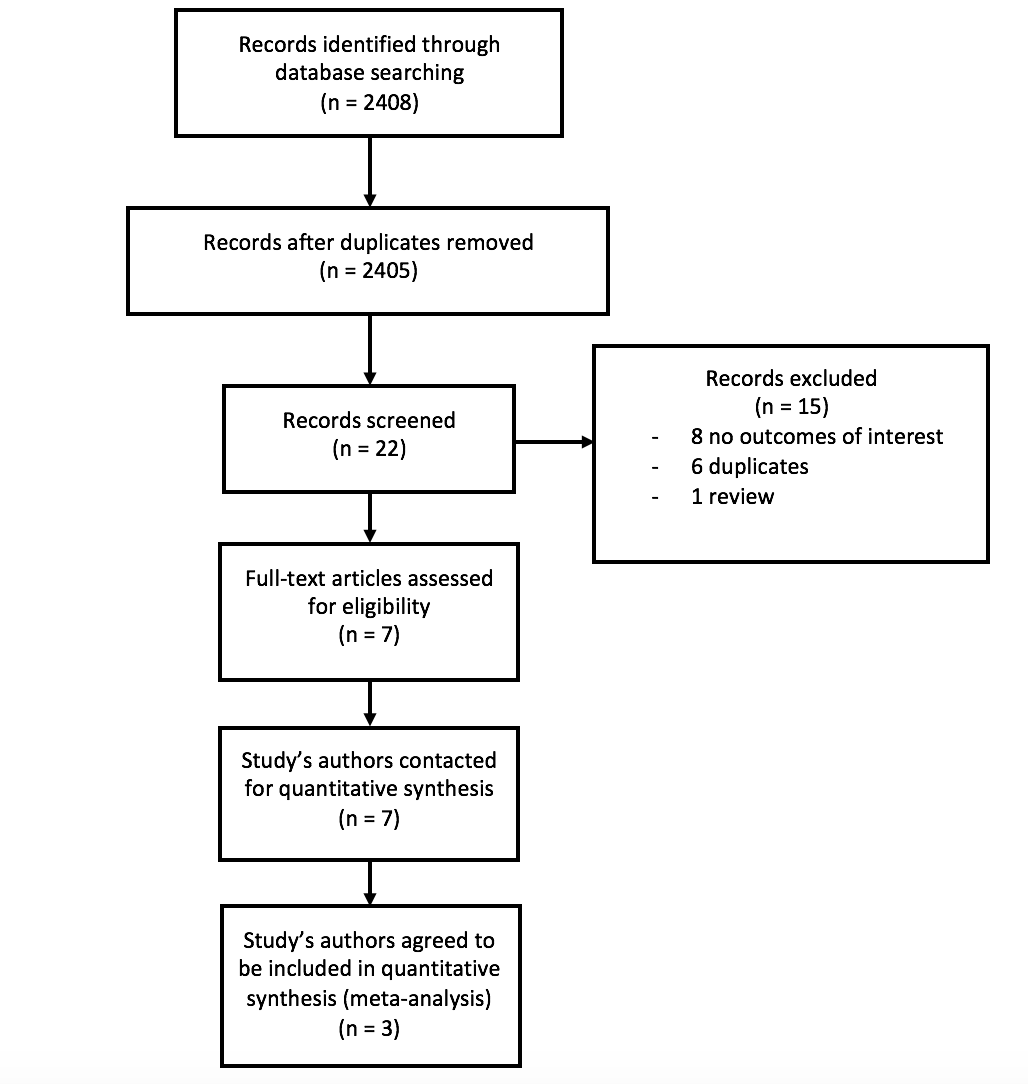
**

**Figure 2s: CHA2DS2-VASc score as predictor of Atrial Fibrillation recurrence – Reciver Operating Characteristic curve.**

AUC: area under the curve


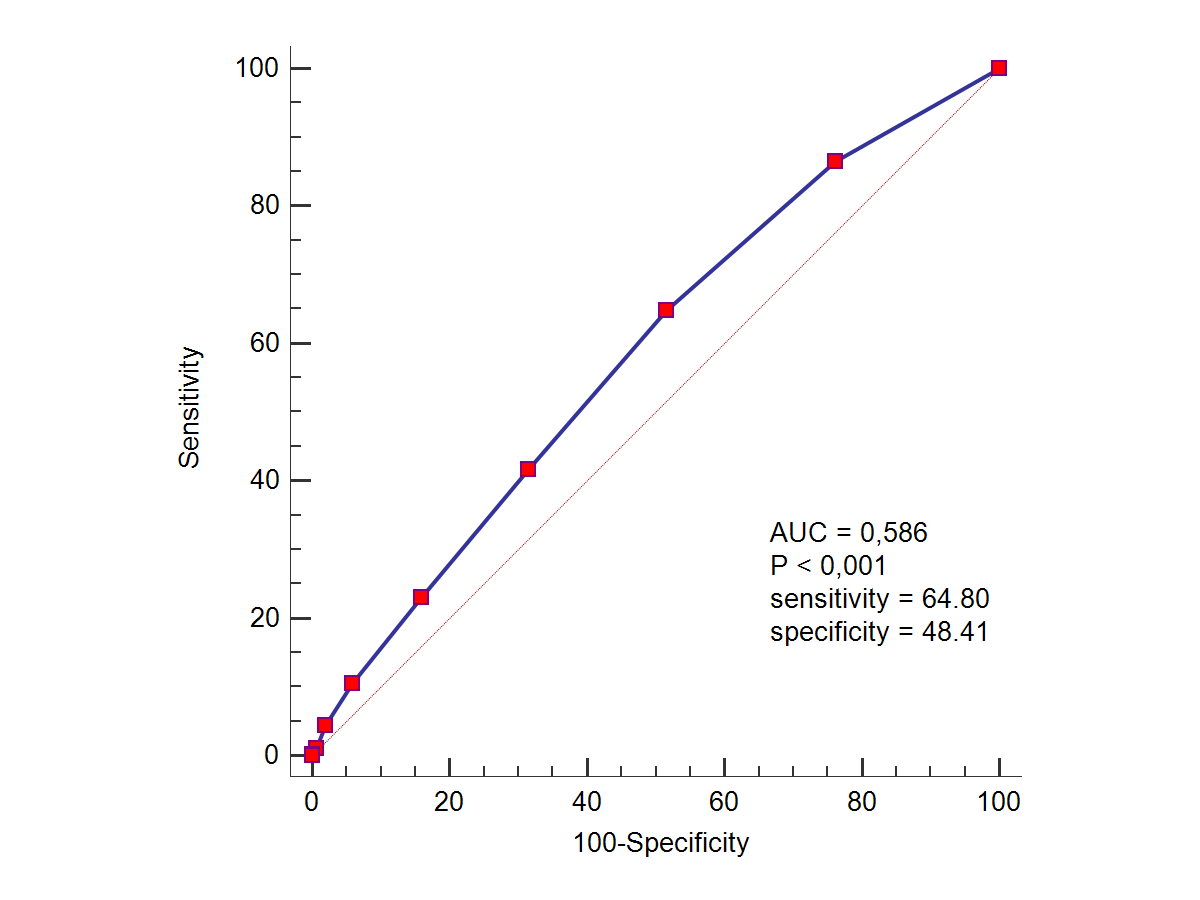

Supplement: Supplementary file 1 — Table S1. New Castle Ottawa scale for quality assessment of paper included in the meta‐analysis Table S2. Studies included in the meta‐analysis Table S3. Univariate logistic regression Figure S1. PRISMA flow‐chart. Figure S2. CHA2DS2‐VASc score as predictor of atrial fibrillation recurrence—receiver operating characteristic curve [file CLC-42-358-s001.docx]
